# Potential Benefits of Acupuncture and Herbs for Obesity-Related Chronic Inflammation by Adipokines

**DOI:** 10.1155/2020/3285363

**Published:** 2020-10-09

**Authors:** Ji-Youn Kim, Seon-Eun Baek, Rehna Paula Ginting, Min-Woo Lee, Jeong-Eun Yoo

**Affiliations:** ^1^Department of Korean Medicine Obstetrics & Gynecology, College of Korean Medicine, Daejeon University, 62, Daehak-ro, Dong-gu, Daejeon 34520, Republic of Korea; ^2^Department of Integrated Biomedical Science and Soonchunhyang Institute of Medi-bio Science (SIMS), Soonchunhyang University, Cheonan-si, Chungcheongnam-do 31151, Republic of Korea

## Abstract

The adipose tissue is an organ that stores energy in the form of fats. It also has been known as an endocrine playing an integral role in metabolic homeostasis by secreting various adipokines. In obesity, the adipokine components and secretion patterns are altered toward proinflammation with weight gain, causing low chronic inflammation, which is closely linked to various metabolic diseases. Acupuncture and herbs are used for the management of obesity and its comorbidities, and it has been observed that these therapies affect the amount of expression and concentration of adipokines with improved metabolic phenotypes in both animal and human metabolic diseases. In this review, we discuss the role of adipokines and summarize beneficial effects of the treatments such as electroacupuncture, pharmacopuncture, catgut embedding acupuncture, and single and multiple medicinal herbs on obesity and its relations to adipokine composition. It will provide a new insight for applying adipokines as surrogate markers in complementary and alternative medicine practice.

## 1. Introduction

The World Health Organization (WHO) defines overweight and obesity as a condition involving accumulation of excess body fat. Specifically, in Western, it regards a body mass index (BMI) equal to or greater than 30 as obese and a BMI of 25 as overweight. According to the WHO statistics, in the US, the percentage of the population with BMI equal to or greater than 30 has increased from 11.9% in 1975 to 36.2% in 2016; during the same period, this percentage has increased from 0.6% to 4.7% in South Korea. These statistics indicate that the prevalence of obesity has increased considerably over the past 40 years [1].

Obesity is an important health issue that causes a wide range of metabolic diseases, necessitating proper treatment and management strategies [2]. In the previous study, complementary and alternative medicine, such as electroacupuncture and catgut embedding acupuncture, represents an efficient therapeutic option for obesity control [3]. Obesity is a low-grade chronic inflammatory disease characterized by an excess accumulation of visceral fat especially white adipose tissue (WAT). Increased WAT mass is known to induce the secretion of proinflammatory mediators including TNF-a and IL-6, resulting in an imbalance in the production of anti-/proinflammatory cytokines [4]. In this review, we searched for studies on acupuncture and herbs regarding to adipokines and presented the main results of English publications obtained from PubMed database and data from works published in Korean language. Those articles which were not relevant to obesity or metabolic disorder were excluded. We accounted for the role of adipokines and introduced several studies regarding the beneficial effects of acupuncture and herbs on metabolic disorders and relations with adipokine composition.

## 2. Chronic Inflammation and Obesity

The development of obesity is closely correlated with the alterations of the immune profile, leading to chronic low-grade inflammation. This state of inflammation is distinguished from general inflammation by different features that the body does not show common signs of inflammation; however, they share similar inflammatory mediators and signaling pathways disorders [5, 6]. Although the issue how obesity causes chronic low-grade inflammation is not yet clear, interesting hypotheses have been proposed. During obesity, adipocytes undergo hypertrophy that leads to an increase of adipose tissue size followed by hypoxia conditions, which contribute to the secretion of proinflammatory adipokines, such as TNF*α*, TGF*β*, IL-6, and MCP-1 by adipocytes and other cells present in adipose tissue [7–13]. The release of these inflammatory adipokines can attract immune cells such as macrophages and T cells to infiltrate into adipose tissue, which is responsible for adipose tissue inflammation [10, 14]. On the other hand, excessive nutrients input triggers an unfolded protein response (UPR) pathway including inositol-requiring enzyme 1 (IRE1), PKR-like endoplasmic-reticulum kinase (PERK), and activating transcription factor 6 (ATF6) in endoplasmic reticulum (ER) that leads to the activation of JNK (c-Jun aminoterminal kinase) and IKK-*β*- (inhibitor of nuclear factor-kB kinase-*β*) dependent inflammatory cascades [15]. Understanding the correlation between obesity and chronic low-grade inflammation can be a potential therapeutic target for the treatment of obesity-related metabolic diseases.

## 3. The Role of Adipokines

### 3.1. Adipose Tissue and Adipokines

White adipose tissue (WAT) is not only an energy storage organ but is also involved in metabolic activities throughout the body by releasing various proteins called adipokines [16, 17]. WAT is primarily distributed beneath the skin and around internal organs. And it is also observed in various organs, including the heart, kidneys, lungs, and adventitia, in an obese individual; metabolic processes associated with these organs are extensively affected by adipokines released from adipose tissues [18, 19]. For example, although leptin and adiponectin released by adipose tissue are mainly known for their roles in appetite and energy metabolism, they also demonstrate a substantial influence on the immune system. Moreover, type 2 diabetes is speculated to develop as these influences block the body's insulin signals to cause insulin resistance. Ongoing research has provided evidence that the process in which obesity causes a metabolic disease is related to the irregular or excessive release of adipokines [20].

For a last decade, novel function of adipocytes has been investigated that unlike WAT, rather dissipate nutrient as heat. Based on their developmental origin and location in body, it is named as brown adipose tissue (BAT) and beige adipose tissue (beige AT). While BAT is usually observed in the interscapular region and kidney and originated from Myf5^+^/PAX7^+^ precursors, beige AT is occurred in subcutaneous adipose tissue under cold or beta-adrenergic stimuli and originated from PDGFRa^+^/SCA1^+^ precursors by de novo differentiation or white adipocyte by transdifferentiation. BAT and beige AT also secrete proteins called batokines, which stimulate and enhance their thermogenic activity through regulation of thermogenic program, immune system, vascularization, and substrate utilization [21].

The pattern of adipokines expression reflects adipose tissue condition or even whole-body energy homeostasis status. The changes in adipokines expression caused by external stimuli or during the development of obesity and T2DM can be a potential marker to explain adipose tissue dysfunction and pathogenic status.

### 3.2. Adipose Tissue and Immunocytes

In obese humans and animals, the number of macrophages greatly increases within the adipose tissue to develop systematic inflammation and insulin resistance [22, 23]. In an individual with normal body weight, the building blocks of adipose tissue include adipocytes, M2 macrophages, CD4+ T cells, and blood vessels; in the adipocytes, anti-inflammatory adipokines such as adiponectin and secreted frizzled-related protein 5 (SFRP5) are preferentially induced, and both vascular function and metabolic regulation remain normal. However, as obesity develops, the size of adipocytes increases; inflammation increases as M1 macrophages and CD8+ T cells spread further and appropriate metabolic regulation becomes challenging. When obesity becomes intractable, it causes comprehensive metabolic dysfunction, during which the following occurs in adipose tissue: adipocytes grow larger; some adipocytes become necrotic; the increased M1 macrophages surround dead adipocytes to form a crown-like structure; the enlarged adipocytes preferentially induce proinflammatory adipokines, including leptin, tumor necrosis factor (TNF), resistin, interleukin (IL)-6, IL-18, retinol-binding protein 4 (RBP4), lipocalin-2, angiopoietin-related protein 2 (ANGPTL-2), C–C motif chemokine ligand 2 (CCL-2), C-X-C motif chemokine 5 (CXCL5), and nicotinamide phosphoribosyl transferase (NAMPT), to cause chronic inflammation and decrease blood circulation to obstruct blood flow [24, 25].

### 3.3. Proinflammatory Adipokines

Leptin is known to regulate the eating behavior via the central nervous system. In leptin-deficient mice (ob/ob mice), bulimia, obesity, and insulin resistance have been documented; these effects were reversed following a leptin injection. However, despite the increased blood leptin concentrations in obese individuals compared to normal subjects, an insufficient anorectic reaction was observed, which could be interpreted as ‘leptin resistance' [26]. Furthermore, leptin increases the production of TNF and IL-6 and promotes the production of CCL-3, CCL-4, and CCL-5, which act as proinflammatory adipokines [27, 28].

In mice, resistin impacts glucose metabolism by blocking insulin signals in adipose tissue [29]; however, its role in glucose metabolism in humans remains unclear [30, 31]. Resistin is synthesized in adipose tissue in mice [32], whereas in humans, it is synthesized primarily in macrophages and monocytes [33]. Resistin synthesized in human monocytes promotes the expression of TNF and IL-6 [34] and inhibits anti-inflammatory reactions of adiponectin in vascular endothelial cells [35, 36].

RBP4 is released by liver cells and transports retinol (vitamin A) throughout the body [37]. Additionally, it is also released by adipose tissue [38] and macrophages [39]. In humans, increased serum RBP4 concentrations increase blood pressure, total cholesterol, neutral fat concentration, and BMI but decrease the high-density lipoprotein (HLD) concentration [40].

Lipocalin 2 (neutrophil gelatinase-associated lipocalin and 24p3) is found in large quantities in adipose tissue [41, 42] and induces an inflammatory response by activating nuclear factor-*κ*B (NF-*κ*B) [43].

ANGPTL2 is a circulating glycoprotein belonging to the angiopoietin-like protein family, which is abundantly expressed in the adipose tissue, lung, kidney, and skeletal muscle. Its circulating levels closely correlate with inflammation and insulin resistance. In obese mice, a decrease in ANGPTL2 attenuated the inflammatory responses and improved insulin resistance. Conversely, increased ANGPTL2 promoted inflammation in adipose tissue and insulin resistance [44].

TNF, a cytokine synthesized mainly by monocytes and macrophages, is known to play an important role in inflammatory responses and autoimmune diseases [45]. Furthermore, TNF is a core substance in the expression of obesity-related insulin resistance, as it promotes insulin resistance by reducing the tyrosine phosphorylation of insulin receptors in muscular and adipose tissues [46].

IL-6 is a proinflammatory cytokine involved in obesity and insulin resistance. In clinical trials, IL-6 has demonstrated a proportional relationship with weight [47, 48].

### 3.4. Anti-Inflammatory Adipokines

Adiponectin, an anti-inflammatory protein hormone, is synthesized only by adipose tissues [16], with serum and adipose tissue levels reportedly lower in obese individuals compared to normal subjects [49]. Among the aforementioned proinflammatory adipokines, TNF and IL-6 interfere with adiponectin synthesis in the adipose tissue [50].

Adiponectin plays an important role in preventing metabolic dysfunctions. In particular, in the body, adiponectin is closely related to the functions of insulin. In an experimental study, when adiponectin was injected into diabetic mice, the efficacy of insulin improved, lowering blood glucose levels [17]. Notably, when 5′ adenosine monophosphate-activated protein kinase (AMPK) is activated, glucose reuptake increases in muscular tissues and gluconeogenesis decreases in the liver. Hence, it appears that insulin functions improve as adiponectin activates AMPK [51, 52].

Additionally, adiponectin inhibits the synthesis of proinflammatory cytokines to prevent metabolic dysfunctions. In patients with obesity or diabetes, the adiponectin concentration in the body was inversely related to the C-reactive protein (CRP) concentration; this was also observed in healthy patients presenting no obesity or diabetes [53]. In serum and adipose tissue, TNF concentrations increased in adiponectin-deficient mice but recovered to normal following the administration of adiponectin [54].

As the adiponectin concentration decreases, the risk of cardiovascular diseases [55], high blood pressure [56], left ventricular hypertrophy [57], and myocardial infarction [58] increases. This is because adiponectin inhibits the expression of TNF and reduces the expression of vascular endothelial cell adhesion molecules (VCAM1), which ultimately leads to a decrease in monocyte adhesion [59–61].

SFRP5 is an anti-inflammatory cytokine that helps improve metabolic dysfunctions, as it prevents the binding of Wnt proteins to receptors. Wnt proteins are closely related to various inflammatory diseases and are primarily expressed in adipose tissue of obese mice. Furthermore, as the degree of obesity increases, the ratio of Wnt5 to SFRP increases. Conversely, SFRP5 is largely observed in the white adipose tissue of healthy mice; it is decreased in adipose tissue of obese mice [62].

## 4. Acupuncture, Herbs, and Adipokines

Traditional Korean medicine is a medical system that has been extensively practiced in Asia, including in China, originating over 2000 years ago, to diagnose, treat, and prevent diseases. In terms of the perspective of traditional Korean medicine, a person is healthy when the yin and yang of the body are balanced but becomes ill when these are imbalanced [63]. Furthermore, the so-called life force energy (qi) and meridians constantly circulate to facilitate the appropriate functioning of body organs, maintaining the body in a stable state. Therefore, when a problem exists in the generation or distribution of life force energy and meridians, body organ functions cannot be adequately maintained, and an individual could develop a disease [64].

In traditional Korean medicine, the causes of obesity include weakened spleen or stomach functions, abnormal body fluid accumulation, insufficient life force energy in the whole body, and the consumption of greasy high-calorific foods. When multiple organ functions are imbalanced, body wastes can excessively accumulate. Particularly, a loss of function of the spleen, lungs, and kidneys, which accumulate abnormal body fluids and wastes, is regarded as the main cause of obesity [65]. To appropriately regulate organ functions, various treatment tools of traditional Korean medicine, such as acupuncture and herbs, are applied. Here, we aimed to comprehensively understand how these treatments influence metabolic disorders and the composition of adipokines (Table 1–4).

### 4.1. Acupuncture

Acupuncture is a medical tool in traditional Korean medicine that prevents, mitigates, and treats diseases by stimulating a certain body surface area to evoke a specific response based on the fundamental theories of the organ function matched meridian system [93].

Electroacupuncture combines acupuncture and electrical stimulation to induce a stronger stimulation. It is often used in obesity treatment for its ability to adjust voltage and maintain a constant frequency [4]. According to a recent study [94], electroacupuncture treatment was effective in weight control and improved the levels of various blood lipids. Furthermore, it has gained attention as a treatment that can be used for the prevention and treatment of obesity-related metabolic syndromes.

Catgut embedding therapy treats a disease by inserting a foreign substance into an acupuncture point or an affected area, providing constant stimulation to the acupuncture point. The advantages include stimulation of a treatment area and maximization of stimulus via long needle-retention time. Recently, it has been widely used in oriental plastic surgery, skincare, and obesity [95].

Pharmacopuncture combines acupuncture and pharmacological therapies, which can stimulate meridian points, producing characteristic drug effects by simultaneously injecting the purified herbal medicine extract into an acupuncture point [96].

#### 4.1.1. Manual Acupuncture

A randomized, controlled trial confirmed that the significant reduction of TNF-*α*, IL-6, and hsCRP in obese patients treated with acupuncture (LI4, LI11, ST21, ST25, ST36, ST40, ST44, SP6, SP15, PC6, LR3, CV4, and CV12, bilaterally, two sessions of 30 minutes/week for six months) [76].

Forty women with a body mass index over 30 were equally randomized to either an acupuncture group or a sham (nonpenetrating) acupuncture group and received treatment at LI4, HT7, ST36, ST44, and SP6 bilaterally. Both groups had two sessions of 20 min/week for a total of 10 sessions. In this study, acupuncture showed its effect on weight loss and its ability to decrease serum insulin and leptin levels and to increase ghrelin and CCK levels [77].

#### 4.1.2. Electroacupuncture

*(1). Animal Experiment*. According to Liaw and Peplow [66], in obese Zucker mice receiving electroacupuncture treatment at 10 Hz (at CV12 and CV4 acupoints for a total of six treatments every other day over two weeks; 0.25 mm × 15 mm in size; electroacupuncture performed for 30 min), the serum TNF-a level was significantly lower than that in untreated mice. However, no significant difference was observed between the two mice groups in the serum leptin, adiponectin, and IL-10 levels. The mean body weight on the first day and last day was decreased in both EA treated group and untreated group. These results are presumed to be the effects of long-term exposure to the anesthetic gas. In the EA treatment group, blood glucose levels in the first and second weeks were similar, whereas in the untreated group, the blood glucose levels were increased in the second week. Therefore, it can be seen that the treatment of EA does not cause hyperglycemia. They also identified that EA increased the serum IL-10 level, the adiponectin : leptin ratio, and IL-10 level in adipose tissue of lean rats [67].

Johansson et al. [68] showed that repeated electrical stimulation regulated key functional molecular pathways important for insulin sensitivity in soleus muscle and mesenteric adipose tissue of DHT-induced PCOS rats. Also, repeated applications of EA were seen to have had a significant differential effect for serum tumor necrosis factor-a, adiponectin, the adiponectin:leptin ratio, and blood glucose in both obese Zucker fatty rats and high-fat diet-induced obese Long Evans rats [69].

Peplow investigated the effect of electroacupuncture at the CV12 and CV4 acupoints in male obese Zucker diabetic rats with different aged mice of 12∼13 weeks and 21∼24 weeks. In young ages, adiponectin and leptin in blood serum and white adipose tissue decreased, while in old ages, no significant differences were noted between the groups in serum insulin, glucose, insulin-to-glucose ratio, adiponectin, leptin, adiponectin-to-leptin ratio, and white adipose tissue [70, 71].

*(2). Clinical Research*. Firouzjaei et al. [78] performed a randomized clinical trial (RCT) to examine the differences in indices such as weight loss and insulin sensitivity between metformin monotherapy and a combined therapy of metformin and acupuncture in overweight/obese patients with type 2 diabetes. In total, 39 patients were recruited: 19 subjects in the case group received metformin and acupuncture and 20 subjects in the control group received metformin and sham acupuncture. Electroacupuncture was performed 10 times, each of which lasted for 30 min, every other day for a total duration of 3 weeks. Electropuncture was performed with a frequency of 15 Hz/10 mA and a needle size of 0.25 mm × 0.40 mm/32 Gauge (EACU brand, Maanshan, Anhui, China), applied to a total of eight points: ST 25, SP 15, ST 28, CV 12, and CV 6. Additionally, ear acupuncture was performed at the following points: Sanjiao, Jidian (hunger), Wei (stomach), Shenmen, Neifenmi (endocrine), and Pi (spleen). In the control group, the needles were inserted superficially at nonacupuncture points in the abdomen, with electric lines connected. For auricular acupuncture, sticky layers without seeds were used in the sham acupuncture therapy. Compared to the control group, in the case group receiving acupuncture and metformin, indices such as body weight, BMI, fasting blood sugar, and the homeostatic model assessment (HOMA) index improved significantly, but serum leptin, resistin, and glucagon-like peptide 1(GLP-1) decreased, and adiponectin and serotonin significantly increased. These results demonstrated that a combination therapy of metformin and acupuncture is effective in anti-inflammatory responses, adipokine improvements, and body weight loss.

#### 4.1.3. Catgut Embedding Acupuncture

*(1) Animal Experiment*. In the study of Yan et al., catgut embedding at points ST 36 and ST 44 promoted weight loss and decreased serum leptin and insulin levels in response to four sessions [72].

*(2) Clinical Research*. Tjan et al. [79] conducted a double blind, randomized, placebo-controlled trial in which 36 obese patients were treated with a combination therapy of dietary intervention and catgut embedding acupuncture, examining the effects of the combination therapy on IL-6 and the BMI. The patients were randomly allocated into two groups, receiving either catgut embedding acupuncture (treatment group) or sham embedding acupuncture (placebo control group). Both groups underwent identical dietary intervention. Overall, two catgut embedding acupuncture sessions were performed over two weeks at the following acupuncture points: CV12, ST25, CV6, and SP6. In the treatment group, IL-6 and BMI decreased. The difference in the average BMI decreased between the two groups and was recorded as 0.34. Regarding the reduced IL-6 levels, no significant difference was observed between the two groups. These results demonstrate that catgut embedding acupuncture lowers IL-6 levels and helps improve BMI in obese patients with diet intervention.

#### 4.1.4. Pharmacopuncture

Nam et al. [97] analyzed clinical and animal experimental research to examine the effects of pharmacopuncture on obesity treatment. Analysis of animal experimental research reported the antiobesity mechanism of pharmacopuncture by documenting the effects of pharmacopuncture on protein concentrations, including TNF-a, IL-6, leptin, and adiponectin. The evaluation of clinical research outlined the relationships between pharmacopuncture and measures such as body weight, waist circumference, and BMI. However, it still remains to investigate the relationship between pharmacopuncture and adipokine in clinical research.

*(1) Animal Experiments*. Jang et al. [73] inserted 0.2 mL of Hagocho (*Prunella vulgaris* L.), Gamgook (*Chrysanthemum indicum* L.), and Galgeun (*Puerariae radix*) at LI11 and ST36, every other day for four weeks, to examine their antiobesity effects. The results demonstrated that Hagocho, Gamgook, and Galgeun decreased the serum concentrations of TNF-a, IL-6, and leptin, plasma glucose, and total cholesterol.

Kim et al. [74] injected wild ginseng complex (WGC), composed of *Panax quinquefolius* (Ginseng Radix Alba: wild American ginseng), *Bos taurus* Linné var. domesticus (Bovis Calculus, ox bezoar), *Ursus arctos* Linné (Fel Ursi, gall bladder of a bear), and *Ondatra zibethicus* (Moschus, musk), into obese mice to examine their antiobesity effects. WGC was injected into the left and right sides of an area that corresponds to BL20 of humans. A dose of 0.2 mL was injected once a day. In the control group, WGC was substituted with physiological saline. The results demonstrated that the WGC injection interfered with the NF-*κ*B expression, which could have inhibited further inflammatory responses. Additionally, in the WGC treatment group, the concentrations of total cholesterol, LDL-cholesterol, and natural fats decreased significantly; the levels of HDL-cholesterol significantly increased; leptin release decreased; and adiponectin expression was enhanced.

Ji et al. [75] injected 0.7 mL *Phaseoli semen* into rats at locations corresponding to human ST36, LI11, and SP6, every other day for four weeks, demonstrating that Phaseoli semen treatment decreased total cholesterol, neutral fats, LDL-cholesterol, TNF-a, and leptin but increased HDL-cholesterol.

### 4.2. Herbal Medicine

#### 4.2.1. Single Herbal Material

Suruga et al. [80] injected a *Rhus verniciflua* leaf extract to high-fat diet-induced obese mice to demonstrate its antiobesity effects. In mice injected with the extract, the rate of weight gain was reduced when compared with the control group mice. Additionally, visceral fat in the abdomen and the serum leptin concentration decreased significantly.

Liao et al. [81] reported that the plasma adiponectin level was greatly increased in diabetic rats treated with *Cirsium japonicum* DC, while no obvious effect of the flavones on the dysregulated plasma insulin level and expressions of leptin and glucose transporter 4 (GLUT4) was observed.

#### 4.2.2. Herbal Formula

*(1) Animal Experiments*. Among the various formulas, several studies have evaluated Bofutsushosan (BOF) (Table 5) [82–85, 92]. Azushima et al. [82] conducted mice experiments to evaluate the mechanisms through which BOF improves different metabolic disorders accompanying obesity. In obese mice injected with BOF, body weight, food intake, LDL-cholesterol, and systolic blood pressure decreased; the concentrations of circulating adiponectin, adiponectin within adipose tissue and PPAR gene expression increased. Lin et al. [83] injected BOF into high-fat-fed mice for four weeks and examined the antidiabetic and antihyperlipidemic effects of BOF by evaluating the blood and musculoskeletal and other tissues. Their results showed that the BOF injection reduced blood glucose and gluconeogenesis in the liver and inhibited serum neutral fats, free fatty acids, and leptin. Akagiri et al. [84] examined the antiobesity effects of BOF in high-fat-fed mice and reported that, in BOF-injected mice, the weight of white adipose tissue was increased and adipose tissue size decreased; the adiponectin concentration in adipose tissue increased; and serum ghrelin concentration decreased. Kobayashi et al. [85] reported that injecting BOF for 25 days to mice fed a high-fat for 5 weeks decreased their body weight, visceral fat, and adipose tissue size and significantly reduced serum concentrations of glucose, insulin, leptin, and natural fats. Additionally, concentrations of adiponectin and leptin and the mRNA expression of uncoupling protein 1 (UCP1) increased in brown adipose tissue.

Hussain et al. [86] and Ansari et al. [87] reported relationships between intestinal microbiota improvement and antiobesity effects of Daesiho-Tang (DSHT) and Chowiseungcheng-tang (CST). In obese mice fed a high-fat diet and treated with DHST, body weight, body fat, total cholesterol, and neutral fat concentration decreased significantly; gene expressions of leptin and adiponectin were regulated; and additionally, intestinal microbiota beneficial to the body increased. In obese mice injected with CST, the expression levels of obesity-related genes were reduced in the hypothalamus; the expression levels of obesity-inhibiting genes were enhanced; the gene expression of an adipokine that promotes obesity, RBP-4, effectively decreased; the gene expression of adiponectin, an adipokine that inhibits obesity, significantly increased; intestinal microbiota beneficial to the body increased in the CST treatment group as observed in the DSHT experiment, Koh et al. [88] injected Yangkyuksanwha-tang (YST) to high-fat diet-induced obese mice for six weeks and reported that the body weight and levels of blood glucose, total cholesterol, neutral fats, and LDL-cholesterol were decreased; HDL-cholesterol was increased; adipose tissues and weights of various organs decreased; the gene expression of leptin decreased; and the gene expression of adiponectin increased. Sung et al. [89] examined the antiobesity, antihyperlipidemic, and antihypertensive effects of Dohaekseunggi-tang (DHSGT) on high-fat diet-induced obese mice. When DHSGT was orally administered to mice, their body weight, liver and adipose tissue mass, size of adipose tissue, and blood pressure decreased when compared to the mice in the control group. DHSGT decreased serum concentrations of total cholesterol, LDL-cholesterol, neutral fats, blood glucose, and leptin, with increased HDL-cholesterol and adiponectin levels observed. Choi et al. [90] examined the antiobesity effects of Taeeumjowui-tang (TJ) on high-fat diet-induced obese mice and demonstrated that the TJ injection improved insulin resistance, dyslipidemia, and hepatic cirrhosis, decreasing serum concentrations of leptin, resistin, and PAI-1 while increasing adiponectin concentration.

Kim et al. [91] examined Tongqiaohuoxue decoction with high-fat diet-induced obese mice. After treatment, proinflammatory cytokines were significantly downregulated in the blood, adipose tissue, and liver. Simultaneously, it significantly reduced plasminogen activator inhibitor-1 (PAI-1) levels in serum, adipose tissue, and the liver.

*(2) Clinical Research*. Azushima et al. [92] conducted a multicenter, randomized, open-label, parallel group controlled trial to investigate the antiobesity and antihypertensive effects of BOF in obese patients with hypertension and observed that the BOF injection decreased systolic blood pressure. Furthermore, while no significant difference was observed in the serum adiponectin concentration between the BOF-injected group and the control group, the resistin concentration increased significantly.

## 5. Perspectives

As the lifestyle and eating habits of modern humans have undergone a dramatic change, the number of obese individuals has increased across all ages. Obesity-related chronic inflammation can give rise to diseases such as type 2 diabetes, dyslipidemia, cardiovascular diseases, and cancer. For the treatment of obesity, Western medicine applies the pharmacological or surgical strategies. Regarding pharmacological treatments, side effects, including lethargy, depression, headache, dizziness, and nausea, may occur [98]. In terms of the surgical interventions used in patients with severe obesity, complications such as central venous sinus thrombosis, pulmonary embolism, pneumonia, myocardial infarction, and cholelithiasis can be followed after surgery [99]. For this reason, there is a need for a noninvasive and safer treatment options.

In the field of traditional Korean medicine, several interventions, including herbal medicine, acupuncture, electroacupuncture, pharmacopuncture, and catgut embedding acupuncture, have been used to treat and manage obesity. These treatments for obesity are to improve the condition in which energy metabolism is imbalanced, problems with the malfunction of the several organs [100]. The adipose tissue is not solely an energy storage organ, but it is also involved in metabolic actions throughout the body by releasing proteins called adipokines. As obesity progresses, proinflammatory adipokines, including leptin, TNF, resistin, IL-6, RBP4, lipocalin-2, and ANGPTL-2, are preferentially synthesized and cause chronic inflammation. Therefore, various traditional Korean medicinal treatments for patients with obesity can be expected to help decrease body weight and related systemic reactions with anti-inflammatory effects, based on which an association to adipokine actions involved in metabolism, immunity, and homeostasis can be inferred.

According to studies that examined adipokine responses to electroacupuncture, pharmacopuncture, and catgut embedding acupuncture, acupuncture treatments decreased TNF-a, leptin, resistin, and IL-6 but increased adiponectin and serotonin. According to studies that examined adipokine responses to single herbal materials and herbal complexes, herbal medicines decreased TNF-a, leptin, resistin, RBP-4, and PAI-1 but increased adiponectin. Electroacupuncture treatment regulated blood glucose and insulin concentrations; catgut embedding acupuncture treatment decreased BMI; various pharmacopuncture treatments were mainly helpful in controlling the cholesterol levels. An *R. verniciflua* leaf extract decreased body weight and visceral fatty tissue; BOF controlled cholesterol levels and decreased blood pressure to express UCP-1, which helped increase the body temperature; DSHT and CST improved intestinal microbiota; YST and DHSGT decreased the size of adipocytes and adipose tissue; TJ improved insulin resistance, dyslipidemia, and hepatic cirrhosis. Traditional Korean medicine treatments that included acupuncture or herbs altered the concentration and expression of obesity-related adipokines and influenced various other indices related to obesity or metabolic diseases.

In this review, we discussed whether obesity treatments utilizing acupuncture and herbal medicine affected adipokines indicating the obesity-related inflammation in animal experiments and clinical research studies. Furthermore, we summarized the changes in adipokines induced by acupuncture or herbal interventions. We suggest that future research requires evaluating the adipokines, which are known to play an integral role in metabolic regulation, in the assessment of obesity treatments utilizing acupuncture and herbs. Moreover, we anticipate that this paper can provide useful basic information for research that aims to examine the effects of acupuncture and herbs on changes in obesity-related inflammatory disease and that aims to search the surrogate markers from adipokines during treatments.

## Figures and Tables

**Table 1:** The summary of acupuncture studies on the changes of metabolic index (animal experiments).

Intervention	Acupoints	Period	Total sessions	Model	Results	References
Electroacupuncture (10 Hz, 15 mA)	CV4 and CV12	For 30 min on alternative weekdays over 2 weeks	6	Male obese Zucker fatty rat	TNF-a↓ and body weight↓	Liaw and Peplow [66]
Electroacupuncture (10 Hz, 15 mA)	CV4 and CV12	On alternate weekdays over 2 weeks	6	Male long Evans rat	Serum IL-10↑, adipose tissue adiponectin : leptin ratio↑, and adipose tissue IL-0↑	Liaw and Peplow [67]
Electrical or manual acupuncture	Abdominal and hind limb muscle	5 times/wk for 4-5 wks	20-25	Female pups	Body weight↓, inguinal fat depot weight↓, and soleus muscle weight↑	Johansson et al. [68]
Electroacupuncture (10 Hz, 15 mA)	CV4 and CV12	On alternate weekdays over 2 weeks	6	Male Zucker fa/fa rat	TNF-a↓, adiponectin↑, adiponectin : leptin ratio↑, and blood glucose↓	Liaw and Peplow [69]
Electroacupuncture (10 Hz, 15 mA)	CV4 and CV12	For 30 min	5	Obese male ZDF rats	Blood glucose↓	P.V. Peplow et al [70]
Electroacupuncture (10 Hz, 15 mA)	CV4 and CV12	For 30 min	6	Obese male ZDF rats	leptin↓, adiponectin : leptin ratio↑, and white adipose tissue↓	P.V. Peplow et al [71]
Catgut embedding acupuncture	ST36 and ST44	Once a week for 4 weeks	4	Male Sprague-Dawley rats	Serum leptin↓, body weight↓, and serum insulin level↓	Yan et al. [72]
Pharmacopuncture (Hagocho, Gamgook, and Galgeun)	LI11 and ST36	Every other day for 4 weeks	14	Male obese rat	TNF-a↓, IL-6↓, leptin↓ plasma glucose↓, total cholesterol↓, FFA↓, triglyceride↓, LDL↓, and HDL↑	Jang et al. [73]
Pharmacopuncture (wild ginseng complex)	BL20	Every day during 6 weeks	42	Male obese rat	leptin↓, adiponectin↑, body weight↓, NF-kB↓, total cholesterol↓, LDL↓, HDL↑	Kim et al. [74]
Pharmacopuncture (Phaseoli semen rubra)	ST36, SI11, and SP6	Every other day for 4 weeks	14	Male obese rat	leptin↓ and no significant difference in TNF-a	Ji et al. [75]

**Table 2:** The summary of acupuncture studies on the changes of metabolic index (clinical research studies).

Study design	Intervention (n)	Comparison (n)	Acupoints	Period	Total sessions	Results	References
RCT	Manual acupuncture (57)	No treatment (23)	LI4, LI11, ST21, ST25, ST36, ST40, ST44, SP6, SP15, PC6, LR3, CV4, and CV12	For 30 min, twice weekly for a month	6	TNF-a↓, IL-6↓, hsCRP↓ total cholesterol↓, triglyceride↓, and fasting blood glucose↓	Ismail et al. [76]
RCT	Manual acupuncture (20)	Sham acupuncture (20)	LI4, HT7, ST36, ST44, and SP6	2 sessions of 20 min/week	10	leptin↓, insulin↓, body weight↓, ghrelin↑, and CCK↑	Güçel et al. [77]
RCT	Electroacupuncture, 15 Hz, 10 mA (19)	Metformin monotherapy (20)	ST25, SP15, ST28, CV4, and CV12	For 30 min on every other day over 3 weeks	10	leptin↓, resistin↓, GLP-1↓, adiponectin↑, serotonin↑, body weight↓, body mass index (BMI)↓, fasting blood sugar (FBS)↓, HOMA index↓, FFAs↓, triglyceride↓, LDL↓, and HDL↑	Firouzjaei et al. [78]
RCT	Catgut embedding acupuncture (18)	Sham (placebo) embedding acupuncture (18)	CV6, CV12, ST25, and SP6	2 weeks interval between procedures	2	IL-6↓, serum glucose↓, total cholesterol↓, FFAs↓, triglyceride↓, LDL↓, and HDL↑	Tjan et al. [79]

**Table 3:** The summary of herbal medicine studies on the changes of metabolic index (animal experiments).

Intervention	Model	Duration	Results	References
Single herbal material				
*Rhus verniciflua* leaf extract	Six-week-old male C57BL/6J mice	56 days	Leptin↓, body weight↓, and intraabdominal fat↓	Suruga et al. [80]
*Cirsium japonicum* DC	Male Sprague-Dawley rats	3 weeks	Adiponectin↑, body weight↓, plasma glucose↓, and plasma triglyceride↓	Liao et a.l [81]
Herbal formula				
Bofu-tsusho-san	Male KKAy mice	8 weeks	Adiponectin↑, body weight↓, food intake↓, LDL↓, and systolic blood pressure↓	Azushima et al. [82]
Bofu-tsusho-san	The C57BL/6J mice	12 weeks	Leptin↓, visceral fat mass↓, hepatic triacylglycerol content↓, and blood glucose level↓	Lin et al. [83]
Bofu-tsusho-san	Male KK/Ta Jcl mice	4 weeks	Adiponectin↑ and weight of WAT↓	Akagiri et al. [84]
Bofu-tsusho-san	Male ICR mice	25 days	Leptin↓, adiponectin↑ body weight↓, total fat mass↓, visceral fat mass↓, ratio of fat mass to body weight↓, and epididymal adipocyte size↓	Kobayashi et al. [85]
Daesiho-tang	Male C57BL/6 mice	12 weeks	Leptin↓, adiponectin↑ body weight↓, total body fat↓, total cholesterol↓, and triglyceride↓	Hussain et al. [86]
Chowiseungcheng-tang	C57BL/6J mice	12 weeks	RBP-4↓, adiponectin↑ body weight↓, food efficiency ratio↓, liver weight↓, and total VAT weight↓	Ansari et al. [87]
Yangkyuksanwha-tang	Male C57bl/6J mice	6 weeks	Leptin↓, adiponectin↑ body weight↓, glucose↓, total cholesterol↓, triglyceride↓, LDL↓, and HDL↑	Koh et al. [88]
Dohaekseunggi-tang	Male C57BL/6J mice	7 weeks	Leptin↓, adiponectin↑ body weight↓, liver and adipose tissue mass↓, adipocyte size↓, blood pressure↓, total cholesterol↓, triglyceride↓, glucose↓, LDL↓, and HDL↑	Sung et al. [89]
Taeeumjowui-tang	C57BL/6J mice	12 weeks	Leptin↓, resistin↓, PAI-1↓, adiponectin↑ body weight↓, food efficiency ratio↓, plasma total cholesterol↓, the hepatic fatty acid, and triglyceride and cholesterol contents↓	Choi et al. [90]
Tongqiaohuoxue decoction	Male C57BL/6 mice	4 weeks	Adiponectin↑, leptin↓, PAI-1↓, blood glucose↓, total cholesterol↓, and triglyceride↓	Kim et al. [91]

**Table 4:** The summary of herbal medicine studies on the changes of metabolic index (clinical research studies).

Study design	Intervention (n)	Comparison (n)	Duration	Results	References
RCT	Bofu-tsusho-san (54)	Conventional control therapy (52)	24 weeks	Resistin↑, body weight↓, body mass index (BMI) ↓, and systolic blood pressure↓	Azushima et al. [92]

**Table 5:** The composition of herbal formula.

Formula name	Pharmacognostic name of herbs	References
Bofu-tsusho-san	*Scutellariae radix*, *Glycyrrhizae radix*, *Platycodi radix*, *Gypsum fibrosum*, *Atractyloids rhizoma*, *Rhei rhizoma*, *Schizonepetae spica*, *Gardeniae fructus*, *Paeoniae radix*, *Cnidii rhizoma*, *Angelicae radix*, *Menthae herba*, *Ledebouriellae radix*, *Ephedrae herba*, *Forsythiae fructus*, *Zingiberis rhizoma*, Kadinum, and *Natrium sulfuricum*	Azushima et al. [82, 92]
Bofu-tsusho-san	*Scutellariae radix*, *Glycyrrhizae radix*, *Platycodi radix*, *Atractyloids lanceae rhizoma*, *Rhei rhizoma*, *Schizonepetae spica*, *Gardeniae fructus*, *Paeoniae radix*, *Cnidii rhizoma*, *Angelicae radix*, *Menthae follium*, *Saposhnikoviae radix*, *Ephedrae herba*, *Forsythiae fructus*, *Zingiberis rhizoma*, *Gypsum fibrosum*, *Natrium sulfuricum,* and *Talcum crystallinum*	Lin et al. [83], Kobayashi et al. [85]
Bofu-tsusho-san	*Scutellariae radix*, *Glycyrrhizae radix*, *Platycodi radix*, *Atractyloids lanceae rhizoma*, *Rhei rhizoma*, *Schizonepetae spica*, *Gardeniae fructus*, *Paeoniae radix*, *Cnidii rhizoma*, *Angelicae radix*, *Menthae follium*, *Saposhnikoviae radix*, *Ephedrae herba*, *Forsythiae fructus*, *Zingiberis rhizoma*, Kaolinum, *Gypsum fibrosum,* and *Natrium sulfuricum*	Akagiri et al. [84]
Daesiho-tang	*Bupleuri radix*, *Pinelliae rhizome*, *Zingiberis rhizome*, *Scutellariae radix*, *Paeoniae radix*, *Zizyphus fructus*, *Ponciri fructus,* and *Rhei undulati rhizome*	Hussain et al. [86]
Chowiseungcheng-tang	*Coicis semen*, *Castaneae semen*, *Raphani semen*, *Ephedrae herba*, *Platycodi radix*, *Liriopis tuber*, *Schizandrae fructus*, *Acori graminei rhizoma*, *Polygalae radix*, *Asparagi radix*, *Zizyphi spinosae semen*, and *Longanae arillus*	Ansari et al. [87]
Yangkyuksanwha-tang	*Rehmannia glutinosa*, *Lonicera japonica* Thunberg, *Forsythia viridissima* Lindley, *Gardenia jasminoides Ellis*, *Mentha arvensis* L. var*. piperascens Malinvaud* ex Holmes, *Anemarrhena asphodeloides* Bunge, *Gypsum*, *Saposhnikovia divaricata* Schischkin, and *Schizonepeta tenuifolia* Briquet	Koh et al. [88]
Dohaekseunggi-tang	*Glycyrrhizae uralensis* Fischer, *Rheum undulatum* Linne, *Prunus persica* Linne, *Cinnamomum cassia* Presl, *and Natrii sulfas*	Sung et al. [89]
Dohongsamul-tang	*Angelis gigantis radix*, *Persicae semen*, *Rehmanniae radix*, *Cnidii rhizome*, and *Carthami flos*	
Taeeumjowui-tang	*Coicis Semen*, *Castaneae Semen*, *Raphani Semen*, *Schizandrae fructus*, *Platycodi radix*, *Radix Ophiopogonis*, *Acori Graminei Rhizoma*, *and Ephedrae herba*	Choi et al. [90]
Tongqiaohuoxue decoction	*Paeonia obovata* Maxim, *Cnidium officinale* Makino, *Prunus persica* (Linne) Batsch, *Carthamus tinctorius* L., *Allium fistulosum* L., *Zizyphus jujube* var. inermis (Bunge) Rehder, *Zingiber officinale* Roscoe, and *Saussurea costus* (Falc.) Lipsch	Kim et al. [91]

## Data Availability

The data used to support the findings of this study are included within the article.

## References

[1] WHO, https://www.who.int/topics/obesity/en/

[2] Jung E. D., Lee J., Shon H.-S. (2007). The correlation between central obesity and glucose, lipid metabolism and macrovascular complication in elderly type 2 diabetes. *The Journal of Korean Diabetes Association*.

[3] Lafontan M. (2014). Adipose tissue and adipocyte dysregulation. *Diabetes & Metabolism*.

[4] Jessica G., Roberto G., Carmen G. (2013). Effects of acupuncture on obesity and adipokines involved in body weight control. *Journal of Homeopathy & Ayurvedic Medicine*.

[5] Castro A. M., Macedo-de la Concha L. E., Pantoja-Meléndez C. A. (2017). Low-grade inflammation and its relation to obesity and chronic degenerative diseases. *Revista Médica del Hospital General de México*.

[6] Stolarczyk E. (2017). Adipose tissue inflammation in obesity: a metabolic or immune response?. *Current Opinion in Pharmacology*.

[7] Surmi B. K., Hasty A. H. (2008). Macrophage infiltration into adipose tissue: initiation, propagation and remodeling. *Future Lipidology*.

[8] Suganami T., Ogawa Y. (2010). Adipose tissue macrophages: their role in adipose tissue remodeling. *Journal of Leukocyte Biology*.

[9] Chan P.-C., Hsieh P.-S., Gordeladze J. The role of adipocyte hypertrophy and hypoxia in the development of obesity-associated adipose tissue inflammation and insulin resistance.

[10] Jo J., Gavrilova O., Pack S. (2009). Hypertrophy and/or hyperplasia: dynamics of adipose tissue growth. *PLOS Computational Biology*.

[11] Halberg N., Khan T., Trujillo M. E. (2009). Hypoxia-inducible factor 1*α* induces fibrosis and insulin resistance in white adipose tissue. *Molecular and Cellular Biology*.

[12] Ye J., Gao Z., Yin J., He Q. (2007). Hypoxia is a potential risk factor for chronic inflammation and adiponectin reduction in adipose tissue of ob/ob and dietary obese mice. *American Journal of Physiology-Endocrinology and Metabolism*.

[13] Chen B., Lam K. S. L., Wang Y. (2006). Hypoxia dysregulates the production of adiponectin and plasminogen activator inhibitor-1 independent of reactive oxygen species in adipocytes. *Biochemical and Biophysical Research Communications*.

[14] Ellulu M. S., Patimah I., Khaza’ai H., Rahmat A., Abed Y. (2017). Obesity and inflammation: the linking mechanism and the complications. *Archives of Medical Science*.

[15] Hotamisligil G. S. (2006). Inflammation and metabolic disorders. *Nature*.

[16] Ouchi N., Kihara S., Funahashi T., Matsuzawa Y., Walsh K. (2003). Obesity, adiponectin and vascular inflammatory disease. *Current Opinion in Lipidology*.

[17] Berg A. H., Scherer P. E. (2005). Adipose tissue, inflammation, and cardiovascular disease. *Circulation Research*.

[18] Samaras K., Botelho N. K., Chisholm D. J., Lord R. V. (2010). Subcutaneous and visceral adipose tissue gene expression of serum adipokines that predict type 2 diabetes. *Obesity*.

[19] Fried S. K., Bunkin D. A., Greenberg A. S. (1998). Omental and subcutaneous adipose tissues of obese subjects release interleukin-6: depot difference and regulation by Glucocorticoid1. *The Journal of Clinical Endocrinology & Metabolism*.

[20] Bluher M., Mantzoros C. S. (2014). From leptin to other adipokines in health and disease: facts and expectations at the beginning of the 21st century. *Metabolism*.

[21] Lee M. W., Lee M., Oh K. J. (2019). Adipose tissue-derived signatures for obesity and type 2 diabetes: adipokines, batokines and MicroRNAs. *Journal of Clinical Medicine*.

[22] Xu H., Barnes G. T., Yang Q. (2003). Chronic inflammation in fat plays a crucial role in the development of obesity-related insulin resistance. *Journal of Clinical Investigation*.

[23] Weisberg S. P., McCann D., Desai M., Rosenbaum M., Leibel R. L., Ferrante A. W. (2003). Obesity is associated with macrophage accumulation in adipose tissue. *Journal of Clinical Investigation*.

[24] Cinti S., Mitchell G., Barbatelli G. (2005). Adipocyte death defines macrophage localization and function in adipose tissue of obese mice and humans. *Journal of Lipid Research*.

[25] Murano I., Barbatelli G., Parisani V. (2008). Dead adipocytes, detected as crown-like structures, are prevalent in visceral fat depots of genetically obese mice. *Journal of Lipid Research*.

[26] Friedman J. M., Halaas J. L. (1998). Leptin and the regulation of body weight in mammals. *Nature*.

[27] Santos-Alvarez J., Goberna R., Sánchez-Margalet V. (1999). Human leptin stimulates proliferation and activation of human circulating monocytes. *Cellular Immunology*.

[28] Kiquchi N., Maeda T., Kobayashi Y. (2009). Leptin enhances CC-chemokine ligand expression in cultured murine macrophage. *Biochem. Biophys. Res. Commun.*.

[29] Steppan C. M., Wang J., Whiteman E. L., Birnbaum M. J., Lazar M. A. (2005). Activation of SOCS-3 by resistin. *Molecular and Cellular Biology*.

[30] Heilbronn L. K., Rood J., Janderova L. (2004). Relationship between serum resistin concentrations and insulin resistance in nonobese, obese, and obese diabetic subjects. *The Journal of Clinical Endocrinology & Metabolism*.

[31] Lee J. H., Chan J. L., Yiannakouris N. (2003). Circulating resistin levels are not associated with obesity or insulin resistance in humans and are not regulated by fasting or leptin administration: cross-sectional and interventional studies in normal, insulin-resistant, and diabetic subjects. *The Journal of Clinical Endocrinology & Metabolism*.

[32] Steppan C. M., Bailey S. T., Bhat S. (2001). The hormone resistin links obesity to diabetes. *Nature*.

[33] Savage D. B., Sewter C. P., Klenk E. S. (2001). Resistin/Fizz3 expression in relation to obesity and peroxisome proliferator-activated receptor- action in humans. *Diabetes*.

[34] Bokarewa M., Nagaev I., Dahlberg L., Smith U., Tarkowski A. (2005). Resistin, an adipokine with potent proinflammatory properties. *The Journal of Immunology*.

[35] Verma S., Li S.-H., Wang C.-H. (2003). Resistin promotes endothelial cell activation. *Circulation*.

[36] Kawanami D., Maemura K., Takeda N. (2004). Direct reciprocal effects of resistin and adiponectin on vascular endothelial cells: a new insight into adipocytokine-endothelial cell interactions. *Biochemical and Biophysical Research Communications*.

[37] Quadro L., Blaner W. S., Salchow D. J. (1999). Impaired retinal function and vitamin A availability in mice lacking retinol-binding protein. *The EMBO Journal*.

[38] Yang Q., Graham T. E., Mody N. (2005). Serum retinol binding protein 4 contributes to insulin resistance in obesity and type 2 diabetes. *Nature*.

[39] Broch M., Ramírez R., Auguet M. T. (2010). Macrophages are novel sites of expression and regulation of retinol binding protein-4 (RBP4). *Physiological Research*.

[40] Graham T. E., Yang Q., Blüher M. (2006). Retinol-binding protein 4 and insulin resistance in lean, obese, and diabetic subjects. *New England Journal of Medicine*.

[41] Yan Q.-W., Yang Q., Mody N. (2007). The adipokine lipocalin 2 is regulated by obesity and promotes insulin resistance. *Diabetes*.

[42] Zhang J., Wu Y., Zhang Y., LeRoith D., Bernlohr D. A., Chen X. (2008). The role of lipocalin 2 in the regulation of inflammation in adipocytes and macrophages. *Molecular Endocrinology*.

[43] Cowland J. B., Muta T., Borregaard N. (2006). IL-1*β*-Specific up-regulation of neutrophil gelatinase-associated lipocalin is controlled by I*κ*B-*ζ*. *The Journal of Immunology*.

[44] Tabata M., Kadomatsu T., Fukuhara S. (2009). Angiopoietin-like protein 2 promotes chronic adipose tissue inflammation and obesity-related systemic insulin resistance. *Cell Metabolism*.

[45] Hotamisligil G., Shargill N., Spiegelman B. (1993). Adipose expression of tumor necrosis factor-alpha: direct role in obesity-linked insulin resistance. *Science*.

[46] Hotamisligil G. S., Budavari A., Murray D., Spiegelman B. M. (1994). Reduced tyrosine kinase activity of the insulin receptor in obesity-diabetes. Central role of tumor necrosis factor-alpha. *Journal of Clinical Investigation*.

[47] Esposito K., Pontillo A., Di Palo C. (2003). Effect of weight loss and lifestyle changes on vascular inflammatory markers in obese women. *JAMA*.

[48] Ziccardi P., Nappo F., Giugliano G. (2002). Reduction of inflammatory cytokine concentrations and improvement of endothelial functions in obese women after weight loss over one year. *Circulation*.

[49] Ryo M., Nakamura T., Kihara S. (2004). Adiponectin as a biomarker of the metabolic syndrome. *Circulation Journal*.

[50] Li S., Shin H. J., Ding E. L., van Dam R. M. (2009). Adiponectin levels and risk of type 2 diabetes. *JAMA*.

[51] Tomas E., Tsao T.-S., Saha A. K. (2002). Enhanced muscle fat oxidation and glucose transport by ACRP30 globular domain: acetyl-CoA carboxylase inhibition and AMP-activated protein kinase activation. *Proceedings of the National Academy of Sciences*.

[52] Yamauchi T., Kamon J., Minokoshi Y. (2002). Adiponectin stimulates glucose utilization and fatty-acid oxidation by activating AMP-activated protein kinase. *Nature Medicine*.

[53] Ouchi N., Kihara S., Funahashi T. (2003). Reciprocal association of C-reactive protein with adiponectin in blood stream and adipose tissue. *Circulation*.

[54] Maeda N., Shimomura I., Kishida K. (2002). Diet-induced insulin resistance in mice lacking adiponectin/ACRP30. *Nature Medicine*.

[55] Sattar N., Wannamethee G., Sarwar N. (2006). Adiponectin and coronary heart disease. *Circulation*.

[56] Iwashima Y., Katsuya T., Ishikawa K. (2004). Hypoadiponectinemia is an independent risk factor for hypertension. *Hypertension*.

[57] Hong S. J., Park C. G., Seo H. S., Oh D. J., Ro Y. M. (2004). Associations among plasma adiponectin, hypertension, left ventricular diastolic function and left ventricular mass index. *Blood Pressure*.

[58] Pischon T., Girman C. J., Hotamisligil G. S. (2004). Plasma adiponectin levels and risk of myocardial infarction in men. *JAMA*.

[59] Ouchi N., Kihara S., Arita Y. (1999). Novel modulator for endothelial adhesion molecules. *Circulation*.

[60] Kobashi C., Urakaze M., Kishida M. (2005). Adiponectin inhibits endothelial synthesis of interleukin-8. *Circulation Research*.

[61] Ouchi N., Kihara S., Arita Y. (2000). Adiponectin, an adipocyte-derived plasma protein, inhibits endothelial NF-*κ*B signaling through a cAMP-dependent pathway. *Circulation*.

[62] Ouchi N., Higuchi A., Ohashi K. (2010). Sfrp5 is an anti-inflammatory adipokine that modulates metabolic dysfunction in obesity. *Science*.

[63] Ernst E. (2006). Methodological aspects of traditional Chinese medicine (TCM). *Annals of the Academy of Medicine, Singapore*.

[64] Yao W., Yang H., Ding G. (2013). Mechanisms of Qi-blood circulation and Qi deficiency syndrome in view of blood and interstitial fluid circulation. *Journal of Traditional Chinese Medicine*.

[65] The Society of Korean Medicine Rehabilitation (2015). *Korean Regabilitation Medicine Ver*.

[66] Liaw J. J. T., Peplow P. V. (2016). Effect of electroacupuncture on inflammation in the obese zucker fatty rat model of metabolic syndrome. *Journal of Acupuncture and Meridian Studies*.

[67] Liaw J. J. T., Peplow P. V. (2016). Effects of electroacupuncture on pro-/anti-inflammatory adipokines in serum and adipose tissue in lean and diet-induced obese rats. *Journal of Acupuncture and Meridian Studies*.

[68] Johansson J., Mannerås-Holm L., Shao R. (2013). Electrical vs manual acupuncture stimulation in a rat model of polycystic ovary syndrome: different effects on muscle and fat tissue insulin signaling. *PLoS One*.

[69] Liaw J. J. T., Peplow P. V. (2016). Differential effect of electroacupuncture on inflammatory adipokines in two rat models of obesity. *Journal of Acupuncture and Meridian Studies*.

[70] Peplow P. V., McLean G. T. Z. (2015). Repeated electroacupuncture: an effective treatment for hyperglycemia in a rat model. *Journal of Acupuncture and Meridian Studies*.

[71] Peplow P. V. (2015). Repeated electroacupuncture in obese zucker diabetic fatty rats: adiponectin and leptin in serum and adipose tissue. *Journal of Acupuncture and Meridian Studies*.

[72] Yan R., Liu X., Bai J., Yu J., Gu J. (2012). Influence of catgut implantation at acupoints on leptin and insulin resistance in simple obesity rats. *Journal of Traditional Chinese Medicine*.

[73] Jang H. J., Lee H. S., Lee J. M. (2007). Effect of Hagocho (*Prunella vularis* L.), Gamgook (*Chrysanthemum indicum* L.) and Galgeun (*Puerariae radix*) aua-acupuncture at gokji (LI 11) and Joksamri (ST 36) on lowering lipid effect, oxidative capacity, concentration of TNF-a, IL-6, leptin and histological consideration in hyperlipidemic rat. *Journal of Acupuncture and Meridian Studies*.

[74] Kim M. W., Lim H. H., Song Y. K. (2012). Anti-obesity effect of Wild Ginseng complex pharmacopuncture on adipocyte and high fat diet-induced obese C57BL/6J mice. *Journal of Rehabilitation Medicine*.

[75] Ji J. H., Lee J. M. (2005). Effects of distilled Phaseoli Semen rubra herbal acupuncture on lipid composition, liver function, anti-oxidant capacity and molecular biological aspects in obese rats induced high fat diet. *Journal of Pharmacopuncture*.

[76] Ismail L. A., Ibrahim A. A., Abdel-Latif G. A. (2015). Effect of acupuncture on body weight reduction and inflammatory mediators in Egyptian obese patients. *Open Access Macedonian Journal of Medical Sciences*.

[77] Güçel F., Bahar B., Demirtas C., Mit S., Çevik C. (2012). Influence of acupuncture on leptin, ghrelin, insulin and cholecystokinin in obese women: a randomised, sham-controlled preliminary trial. *Acupuncture in Medicine*.

[78] Firouzjaei A., Li G. C., Wang N. (2016). Comparative evaluation of the therapeutic effect of metformin monotherapy with metformin and acupuncture combined therapy on weight loss and insulin sensitivity in diabetic patients. *Nutrition & Diabetes*.

[79] Tjan P. M., Srilestari A., Abdurrohim K. (2017). Combination therapy efficacy of catgut embedding acupuncture and diet intervention on interleukin-6 levels and body mass index in obese patients. *Journal of Physics: Conference Series*.

[80] Suruga K., Tomita T., Kadokura K. (2019). Rhus verniciflua leaf extract suppresses obesity in high-fat diet-induced obese mice. *Food & Nutrition Research*.

[81] Liao Z., Chen X., Wu M. (2010). Antidiabetic effect of flavones from Cirsium japonicum DC in diabetic rats. *Archives of Pharmacal Research*.

[82] Azushima K., Tamura K., Wakui H. (2013). Bofu-tsu-shosan, an oriental herbal medicine, exerts a combinatorial favorable metabolic modulation including antihypertensive effect on a mouse model of human metabolic disorders with visceral obesity. *PLoS One*.

[83] Lin C.-H., Kuo Y.-H., Shih C.-C. (2014). Effects of Bofu-Tsusho-San on diabetes and hyperlipidemia associated with AMP-activated protein kinase and glucose transporter 4 in high-fat-fed mice. *International Journal of Molecular Sciences*.

[84] Akagiri S., Naito Y., Ichikawa H. (2008). Bofutsushosan, an oriental herbal medicine, attenuates the weight gain of white adipose tissue and the increased size of adipocytes associated with the increase in their expression of uncoupling protein 1 in high-fat diet-fed male KK/Ta mice. *Journal of Clinical Biochemistry and Nutrition*.

[85] Kobayashi S., Kawasaki Y., Takahashi T. (2017). Mechanisms for the anti-obesity actions of bofutsushosan in high-fat diet-fed obese mice. *Chinese Medical Journal*.

[86] Hussain A., Yadav M. K., Bose S. (2016). Daesiho-tang is an effective herbal formulation in attenuation of obesity in mice through alteration of gene expression and modulation of intestinal microbiota. *PLoS One*.

[87] Ansari A., Bose S., Yadav M. K. (2016). CST, an herbal formula, exerts anti-obesity effects through brain-gut-adipose tissue Axis modulation in high-fat diet fed mice. *Molecules*.

[88] Koh Y. M., Jang S. W., Ahn T. W. (2019). Anti-obesity effect of Yangkyuksanwha-tang in high-fat diet-induced obese mice. *BMC Complementary and Alternative Medicine*.

[89] Sung Y. Y., Kim D. S., Choi G. Y. (2014). Dohaekseunggi-tang extract inhibits obesity, hyperlipidemia, and hypertension in high-fat diet-induced obese mice. *BMC Complementary and Alternative Medicine*.

[90] Choi J. Y., Kim Y. J., Cho S. J. (2017). Metabolic effect of an oriental herbal medicine on obesity and its comorbidities with transcriptional responses in diet-induced obese mice. *International Journal of Molecular Sciences*.

[91] Kim S.-H., Park H.-S., Hong M. J. (2016). Tongqiaohuoxue decoction ameliorates obesity-induced inflammation and the prothrombotic state by regulating adiponectin and plasminogen activator inhibitor-1. *Journal of Ethnopharmacology*.

[92] Azushima K., Tamura K., Haku S. (2015). Effects of the oriental herbal medicine Bofu-tsusho-san in obesity hypertension: a multicenter, randomized, parallel-group controlled trial (ATH-D-14-01021.R2). *Atherosclerosis*.

[93] Korean Acupuncture & Moxibustion Medicine Society Textbook Compilation Committee (2012). *The Acupuncture and Moxibustion Medicine*.

[94] Jiao L., Chi Z. H. (2008). Electroacupuncture for treatment of simple obesity complicated with fatty liver. *Zhongguo Zhen Jiu*.

[95] Huang C.-Y., Choong M.-Y., Li T.-S. (2012). Treatment of obesity by catgut embedding: an evidence-based systematic analysis. *Acupuncture in Medicine*.

[96] Kim M. W., Song Y. K., Lim H. H. (2011). Study of experimentations and clinical trials’ trends for obesity treatment using pharmacopuncture. *Journal of Korean Oriental Association for Study of Obesity*.

[97] Nam M.-H., Lee S.-W., Na H.-Y. (2016). Herbal acupuncture for the treatment of obesity. *Journal of Acupuncture and Meridian Studies*.

[98] Kang J. G., Park C.-Y. (2012). Anti-obesity drugs: a review about their effects and safety. *Diabetes & Metabolism Journal*.

[99] Lee H. C. (2012). Anti complications of surgery for morbid obesity: laparoscopic adjustable gastric banding. *Journal of Metabolic and Bariatric Surgery*.

[100] Lee J. S., Lee S. H. (2001). The reductive effects of oriental medicine on the body fat and abdominal obesity. *Journal of Korean Medicine for Obesity Research*.

